# Isocitrate lyase plays important roles in plant salt tolerance

**DOI:** 10.1186/s12870-019-2086-2

**Published:** 2019-11-06

**Authors:** Worawat Yuenyong, Supaart Sirikantaramas, Li-Jia Qu, Teerapong Buaboocha

**Affiliations:** 1 Molecular Crop Research Unit, Department of Biochemistry, Faculty of Science, Chulalongkorn University, Bangkok, 10330 Thailand; 2 Omics Sciences and Bioinformatics Center, Faculty of Science, Chulalongkorn University, Bangkok, 10330 Thailand; 3 State Key Laboratory for Protein and Plant Gene Research, Peking-Tsinghua Center for Life Sciences at College of Life Sciences, Peking University, Beijing, 100871 China; 4 The National Plant Gene Research Center (Beijing), Beijing, 100101 China

**Keywords:** Isocitrate lyase, Calmodulin, *OsCam1–1*, *OsICL*, Salt stress

## Abstract

**Background:**

Isocitrate lyase (ICL) is a key enzyme in the glyoxylate cycle. In a previous study in rice, the expression of the ICL-encoding gene (*OsICL*) was highly induced by salt stress and its expression was enhanced in transgenic rice lines overexpressing *OsCam1–1*, a calmodulin (CaM)-encoding gene. CaM has been implicated in salt tolerance mechanisms in plants; however, the cellular mechanisms mediated by CaM are not clearly understood. In this study, the role of *OsICL* in plant salt tolerance mechanisms and the possible involvement of CaM were investigated using transgenic plants expressing *OsICL* or *OsCam1–1*.

**Results:**

*OsICL* was highly expressed in senesced leaf and significantly induced by salt stress in three *OsCam1–1* overexpressing transgenic rice lines as well as in wild type (WT). In WT young leaf, although *OsICL* expression was not affected by salt stress, all three transgenic lines exhibited highly induced expression levels. In Arabidopsis, salt stress had negative effects on germination and seedling growth of the *AtICL* knockout mutant (*Aticl* mutant). To examine the roles of *OsICL* we generated the following transgenic Arabidopsis lines: the *Aticl* mutant expressing *OsICL* driven by the native *AtICL* promoter, the *Aticl* mutant overexpressing *OsICL* driven by the 35SCaMV promoter, and WT overexpressing *OsICL* driven by the 35SCaMV promoter. Under salt stress, the germination rate and seedling fresh and dry weights of the *OsICL*-expressing lines were higher than those of the *Aticl* mutant, and the two lines with the *icl* mutant background were similar to the WT. The *F*_v_/*F*_m_ and temperature of rosette leaves in the *OsICL*-expressing lines were less affected by salt stress than they were in the *Aticl* mutant. Finally, glucose and fructose contents of the *Aticl* mutant under salt stress were highest, whereas those of *OsICL*-expressing lines were similar to or lower than those of the WT.

**Conclusions:**

*OsICL*, a salt-responsive gene, was characterized in the transgenic Arabidopsis lines, revealing that *OsICL* expression could revert the salt sensitivity phenotypes of the *Aticl* knockout mutant. This work provides novel evidence that supports the role of ICL in plant salt tolerance through the glyoxylate cycle and the possible involvement of *OsCam1–1* in regulating its transcription.

## Background

Salinity is a major abiotic constraint for plants because it causes osmotic and ionic stresses. Plant responses to these stresses involve complex molecular and biochemical mechanisms. Salinity negatively affects plants in many ways, including inhibition of growth, development, photosynthesis, and yield [1, 2]. Plants have systematic adaptation processes to cope with salt stress, starting with stress perception mechanisms, through signaling cascades and regulation of gene expression, to physiological responses. Previously characterized acclimation processes include osmolyte accumulation to adjust osmotic pressure in roots, morphology adaptation of roots to minimize the root surface, stomata closure to reduce water loss, elimination of excess Na^+^ via extrusion, compartmentation and reabsorption processes, and antioxidation systems [3]. These processes require a large amount of energy from respiration, which needs to be conducted through cost-efficient strategies so the plants can maintain the ability to grow, through more slowly, and produce a harvestable yield [4].

Ca^2+^ has long been known as a major second messenger in signal transduction pathways of eukaryotic cell. Plants use calcium signaling to perceive and respond to environmental stimuli, including abiotic stress [5]. The calcium-signaling pathway is involved in alterations of Ca^2+^ concentrations in the cytosol that result from the release of Ca^2+^ from intracellular organelles or from outside the cell [5, 6]. First, the membrane receptor recognizes the stress signal, resulting in activation of phospholipase C. The activated phospholipase C hydrolyses phosphatidylinositol-4,5-bisphosphate to inositol-1,4,5-trisphosphate, which mediates an increase in the concentration of cytoplasmic Ca^2+^. The signal is perceived by Ca^2+^ sensors, which enhances downstream effects via, for example, kinases or phosphatases that regulate the expression of stress responsive genes, leading to adaptive physiological responses [7, 8]. Over 250 calcium sensor proteins have been identified in Arabidopsis, and they have been categorized in three major families: calmodulin (CaM) and calmodulin-like proteins, calcineurin-B-like proteins, and calcium-dependent protein kinases and calcium- and calmodulin-dependent protein kinases [9].

Our previous transcriptomic analysis showed that the rice isocitrate lyase gene (*OsICL*) was induced by salt stress, and that its expression was enhanced by the combined effect of salt stress and overexpression of a rice calmodulin gene (*OsCam1–1*) [10]. Isocitrate lyase (ICL) is a key enzyme in the glyoxylate cycle, which is the bypassed pathway of the TCA cycle that converts isocitrate to glyoxylate and succinate. In Arabidopsis during germination, ICL plays an important role in lipid-sugar conversion using the acetyl unit from acetyl-CoA, the product of β-oxidation, via the glyoxylate cycle and gluconeogenesis [11, 12]. *ICL* is a single-copy gene in both rice [13] and Arabidopsis [14]. The nucleotide sequences of the *ICL* genes and the encoded amino acid sequences of rice and Arabidopsis share 66.07 and 72.50% identities, respectively. Cooper and Beevers [15] studied mitochondria and glyoxysomes of castor bean endosperm and found that more than 85% of the enzyme activity in the glyoxysomes was from ICL and malate synthase, another key enzyme in the glyoxylate cycle. ICL and malate synthase were found to be involved in the transition of leaf peroxisomes to glyoxysomes, and this process was correlated with senescence [16].

Because the CaM action involves many downstream components that are spread widely in cells, the roles of CaM in conjunction with these components have not been completely understood, especially the roles of CaM in salt stress responses. Although CaM has been shown to contribute to the salt stress tolerance of plants [17, 18], the actual mechanisms have not been characterized. Our previous study showed that *OsICL* expression was highly induced by *OsCam1–1* overexpression [10], however, to our knowledge, there are no clear evidence showing that ICL facilitates plant salt tolerance. Therefore, the roles of *OsICL* and CaM in salt tolerance and CaM involvement were investigated in this study.

## Results

### Induced *OsICL* expression in rice lines overexpressing *OsCam1–1* under salt stress

**Fig. 1:**
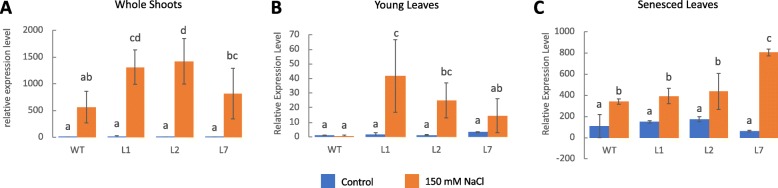
**a** qRT-PCR analysis showing *OsICL* gene expression levels in the whole shoots, **b** young leaves and **c** senesced leaves of the three transgenic rice over-expressing *OsCam1–1* (L1, L2, L7) and WT under non-stress and salt stress at 150 mM NaCl for 4 h (*N* = 4). The relative expression of the whole shoots was compared using whole shoots of WT under non-stress as base line, and the relative expression level of the young and senesced leaves was compared using young leaves under non-stress as baseline. One-way ANOVA with Duncan multiple range test with criteria of *p* < 0.05 was separately applied for statistical data analysis of whole shoots, young leaves, and senesced leaves. Data are shown as the mean ± 1 SD, and means with the same letter are not significantly different

To verify our previously reported transcriptome results of the transgenic rice overexpressing *OsCam1–1* [10], transcript expression levels of *OsICL* in 3-week old seedling shoots of three independent transgenic lines, L1, L2, and L7, were examined by qRT-PCR. We used the 2^-(ΔΔCT)^ method to analyze the relative changes in gene expression with the expression of *OsICL* in the wild type (WT) rice under non-stress condition as the baseline. The expression levels of *OsICL* in WT and in the three transgenic lines under the non-stress condition were not different. Under the salt stress condition, *OsICL* expression levels in the three transgenic lines increased sharply, and were much higher than in the WT (Fig. 1a). The *OsICL* expression levels increased by about 500-fold in the WT, and by about 1300-, 1400-, and 800-folds in L1, L2, and L7, respectively, compared with the baseline.

We determined the expression levels of *OsICL* in WT and in the three transgenic rice lines using first leaf (lowest leaf) to represent a senesced leaf and the third leaf to represent a young leaf. Under the non-stress condition, the senesced leaves showed significantly higher *OsICL* expression levels than the young leaves. Under salt stress condition, the *OsICL* expression levels increased in both leaves, but the senesced leaves still showed higher *OsICL* expression levels than the young leaves (Fig. 1b, c). In WT, L1, L2, and L7, the fold changes in senesced leaves were about 340, 390, 435, and 800 over the baseline of the WT young leaf under the non-stress condition. However, when compared with the WT senesced leaf under salt stress these changes were statistically significant only for L7. Interestingly, the young leaves from the three transgenic lines showed increased *OsICL* expression levels with fold changes in L1, L2, and L7 of about 40, 25, and 15 over the baseline, whereas there was no such change in the young leaf from WT (Fig. 1b).

**Fig. 2:**
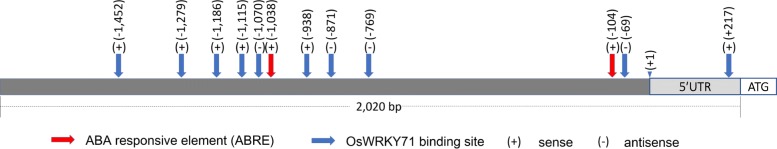
Cis-acting elements of a 2020-bp upstream sequence of *OsICL*. The numbers indicate position of the sites on the sequence

To examine the promoter sequence of *OsICL*, we used the 2020-bp upstream sequence, which included a predicted 5′UTR of 248 bp from the Phytozome database [19] and analyzed it using PLACE, a web-based tool for analyzing cis-acting regulatory DNA elements [20]. Nine OsWRKY71 (LOC_Os02g08440) binding regions were predicted upstream of the 5′UTR and one OsWRKY71 binding region was predicted in the 5′UTR. In addition, two abscisic acid (ABA) responsive elements (ABRE) were found upstream of the 5′UTR (Fig. 2).

### Generation and verification of transgenic Arabidopsis expressing *OsICL*

Three transgenic Arabidopsis lines expressing *OsICL* were generated successfully: 3FL9, the *Aticl* Arabidopsis mutant expressing *OsICL* driven by the 2138-bp upstream sequence of the *AtICL* gene; OX*OsICL*/*icl*, the *Aticl* mutant expressing *OsICL* driven by the 35SCaMV promoter; and OX*OsICL*/WT, WT Arabidopsis expressing *OsICL* driven by the 35SCaMV promoter. PCR and agarose gel electrophoresis confirmed construction of the recombinant plasmids (Additional file 1A and B) and the background genotypes of all five plants examined: 3FL9, OX*OsICL*/*icl*, OX*OsICL*/WT, *Aticl* mutant, and WT (Additional file 1C) and the *OsICL* gene insertion in all *OsICL*-expressing transgenic Arabidopsis lines (Additional file 1D).

The qRT-PCR analysis showed that *OsICL* was expressed in the three transgenic Arabidopsis containing the gene construct but not in the WT and *Aticl* mutant under both non-stress and salt stress conditions (Table 1). Among the three transgenic Arabidopsis lines, OX*OsICL*/WT exhibited outstandingly high *OsICL* expression levels under both the non-stress and salt stress conditions; however, the *OsICL* expression level was significantly higher under salt stress than under the non-stress condition. For the two transgenic Arabidopsis lines, 3FL9 and OX*OsICL*/*icl*, with the *Aticl* mutant background, the *OsICL* expression levels were lower than they were for OX*OsICL*/WT, but its *OsICL* expression levels were higher for OX*OsICL*/*icl* than for 3FL9. Expression of the native Arabidopsis isocitrate lyase gene (*AtICL)* was also determined, and the results showed that, under salt stress, *AtICL* expression was detected only in WT and OX*OsICL*/WT, the two lines with the WT background (Table 2).

**Table 1:** Expression levels obtained by qRT-PCR of *OsICL* in 10-day-old transgenic Arabidopsis seedlings

Lines	2^-(∆CT)^ ± SD	
	Control	120 mM NaCl
wild type	ND	ND
*Aticl* mutant	ND	ND
3FL9	0.0247 ± 0.0040^a^	0.0077 ± 0.0030^a^
OX*OsICL*/*icl*	0.1251 ± 0.0391^a^	0.0745 ± 0.0194^a^
OX*OsICL*/WT	3.6645 ± 0.6099^b^	5.2608 ± 0.9758^c^

One-way ANOVA with Duncan multiple range test was used for the data analysis

Data are shown as the mean ± 1 SD, and different lowercase letters indicate significant difference among plant lines under both control and stress conditions (*p* < 0.05)

ND, no *OsICL* expression was detected

**Table 2:** Expression levels obtained by qRT-PCR of *AtICL* in 10-day-old Arabidopsis seedlings

Lines	2^-(∆CT)^ ± SD	
	Control	120 mM NaCl
wild type	ND	0.0027 ± 0.0015^b^
*Aticl* mutant	ND	ND
3FL9	ND	ND
OX*OsICL*/*icl*	ND	ND
OX*OsICL*/WT	ND	0.0003 ± 0.0002^a^

One-way ANOVA with Duncan multiple range test was used for the data analysis

Data are shown as the mean ± 1 SD, and different lowercase letters indicate significant difference (*p* < 0.05)

ND, no *AtICL* expression was detected

**Fig. 3:**
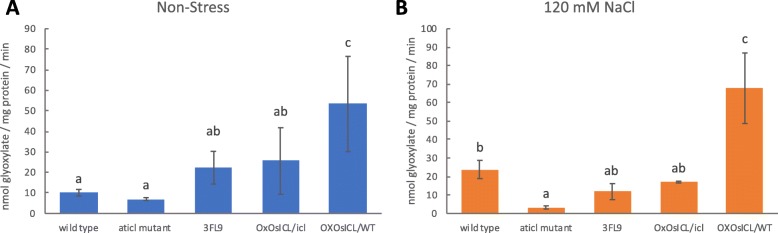
Isocitrate lyase activity of the five Arabidopsis lines **a** under normal condition, and **b** under salt stress at 120 mM NaCl for 10 days (*N* = 3). One-way ANOVA with Duncan multiple range test (*p* < 0.05) was used in the isocitrate lyase activity data analysis. Data are shown as the mean ± 1 SD, and means with the same letter are not significantly different

The activity of ICL was determined using an assay that measured the amount of glyoxylate production. The highest ICL activity was found in OX*OsICL*/WT under both the non-stress and salt stress conditions (Fig. 3). Under the non-stress condition, ICL activity was higher in 3FL9 and OX*OsICL*/*icl* than the WT and *Aticl* mutant, however, under salt stress, ICL activity increased in WT but not in the *Aticl* mutant and other lines with the *Aticl* mutant background.

### Transgenic Arabidopsis expressing *OsICL* exhibited better germination and seedling growth under salt stress than the *Aticl* mutant

**Fig. 4:**
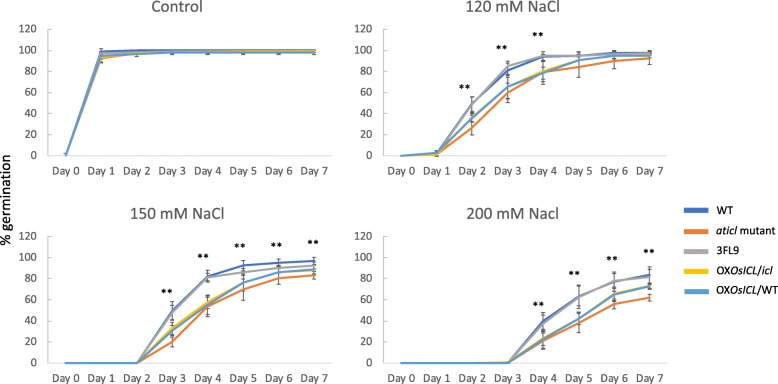
Germination rate of the five Arabidopsis lines (*N* = 5). One-way ANOVA was used to compare percentage of germination in each day separately, with five biological replicates. The error bars represent SD and the two asterisks represent statistically significant difference with *p* < 0.01

Under the non-stress condition, all Arabidopsis lines almost completely germinated after 1 day (Fig. 4). Under 120 mM NaCl stress condition, WT and 3FL9 had the highest germination rates whereas the *Aticl* Arabidopsis mutant had the lowest germination rate. Interestingly, although the two transgenic Arabidopsis lines overexpressing *OsICL* under the control of the 35SCaMV promoter, OX*OsICL*/WT and OX*OsICL*/*icl*, had lower germination rate than the WT and 3FL9, they had higher germination rates under salt stress than the *Aticl* mutant, and this was more pronounced at higher NaCl concentrations.

**Fig. 5:**
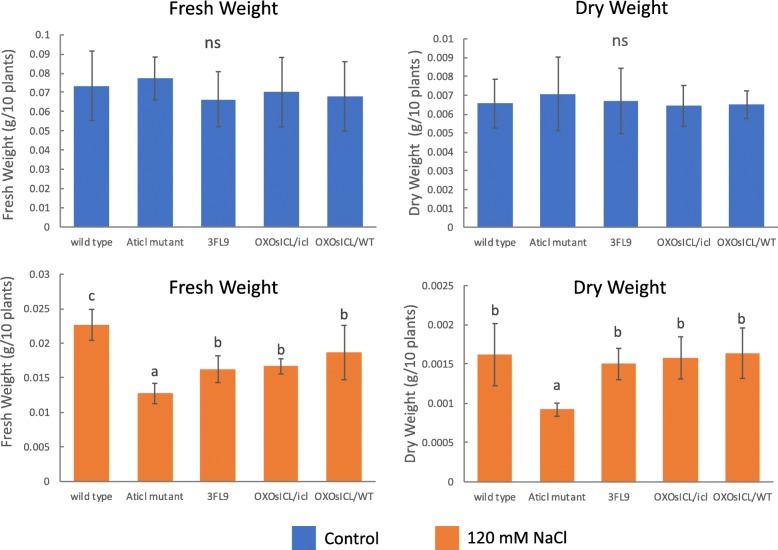
Fresh weight and dry weight of the five Arabidopsis lines (*N* = 5), One-way ANOVA with Duncan multiple range test (*p* < 0.05) was used to analyze the fresh weight and dry weight data with five biological replicates. Data are shown as the mean ± 1 SD, and means with the same letter are not significantly different

**Fig. 6:**
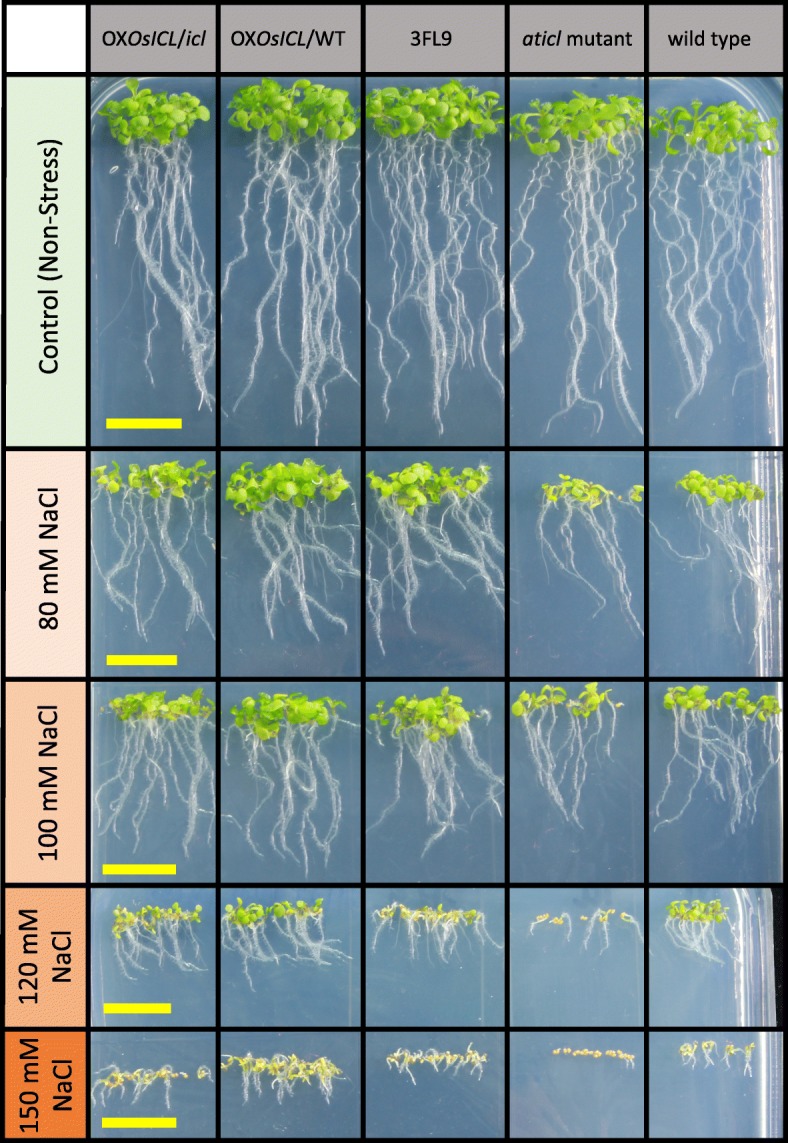
The five Arabidopsis lines growing in MS medium containing various NaCl concentrations at day 10 of growth. The scale bars represent 1 cm

To investigate the effect of *OsICL* expression levels on seedling growth under salt stress, the fresh and dry weights of the five Arabidopsis lines were determined. The results showed that under the non-stress condition the fresh weights and dry weights of the five Arabidopsis lines were not significantly different. Under salt-stress, the changes in the fresh and dry weights had a similar pattern, in which the *Aticl* mutant had significantly lower fresh and dry weighs than the other Arabidopsis lines (Fig. 5). Furthermore, by appearance, the *Aticl* mutant showed growth defect more frequently than the other Arabidopsis lines under salt stress, particularly under high salt concentration (Fig. 6).

### Transgenic Arabidopsis expressing *OsICL* exhibited less salt stress effects than the *Aticl* mutant

**Fig. 7:**
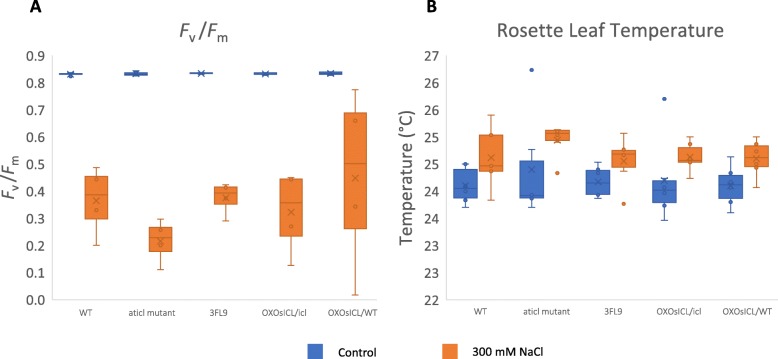
Box-plot chart showing **a** *F*_v_/*F*_m_ of rosette leaf under non-stress or salt stress at 300 mM NaCl for 3 days (*N* = 4), **b** Arabidopsis rosette leaf temperature under non-stress or salt stress at 300 mM NaCl for 3 days (*N* = 6), with the cross symbol represents mean

To examine the effect of salt stress on photosynthesis, *F*_v_*/F*_m_, which indicates maximum potential quantum efficiency of Photosystem II in the dark-adapted state [21], was measured using a pocket chlorophyll fluorimeter. The results showed that under the non-stress condition there were no difference in *F*_v_*/F*_m_ in the five Arabidopsis lines (Fig. 7a). Under salt stress, the *F*_v_*/F*_m_ decreased significantly in the five Arabidopsis lines; the *Aticl* mutant had the lowest *F*_v_*/F*_m_ values and OX*OsICL*/WT had the highest. Rosette leaf temperature of 4-week-old transgenic Arabidopsis lines expressing *OsICL*, WT, and the *Aticl* mutant was measured after non-stress and 300 mM NaCl stress for 3 days. Under the non-stress condition, no differences were detected in the temperatures in the five Arabidopsis lines (Fig. 7b and Additional file 2). Under salt stress, the temperature increased in all five lines, especially in the *Aticl* mutant, which had highest rosette leaf temperature. Overall, the changes in *F*_v_*/F*_m_ and rosette leaf temperature indicated that the *Aticl* mutant was the most affected by salt stress among the five Arabidopsis lines examined and *OsICL* expression reverted these salt sensitivity phenotypes of the *Aticl* knockout mutant.

### Effect on sugar content of *OsICL* expression in Arabidopsis under salt stress

**Fig. 8:**
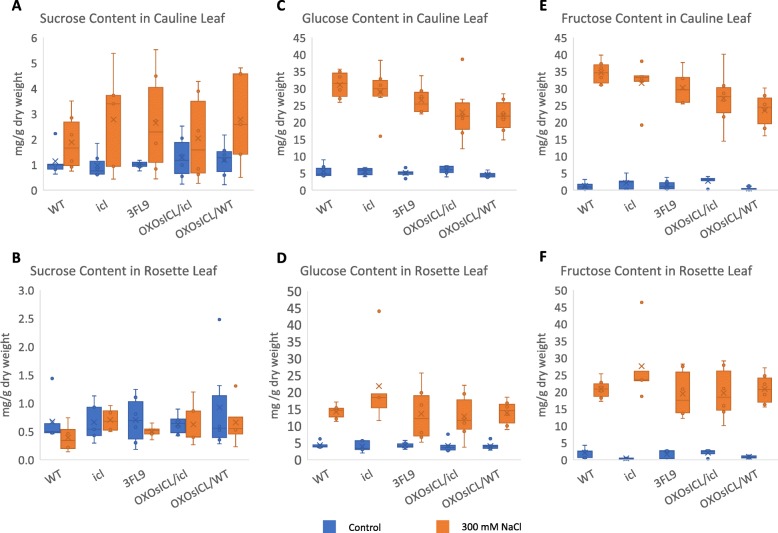
Box-plot chart showing sucrose content of **a** cauline leaf, **b** rosette leaf; glucose content of **c** cauline leaf, **d** cauline leaf; and fructose content of **e** cauline leaf, **f** cauline leaf under non-stress or salt stress at 300 mM NaCl, with the cross symbol represents mean (*N* = 6)

The role of ICL in the plant glyoxylate cycle involves the bypass of the TCA and the conversion of lipid to sugar via a gluconeogenesis process, so we determined the sucrose, glucose, and fructose contents in cauline and rosette leaves of 4-week-old transgenic Arabidopsis lines expressing *OsICL*, *Aticl* mutant, and WT, under non-stress and 300 mM NaCl stress. We found that the sucrose content of cauline leaf but not rosette leaf increased under salt stress in all five Arabidopsis lines (Fig. 8a, b), whereas, the glucose and fructose contents increased significantly in both cauline and rosette leaves under the same salt stress condition (Fig. 8c-f). Surprisingly, the glucose and fructose contents of the cauline and rosette leaves in the *Aticl* mutant under salt stress were one of the highest comparing among the five Arabidopsis lines. In cauline leaf, the glucose and fructose contents of the *OsICL*-expressing Arabidopsis lines, especially OX*OsICL*/WT, under salt stress were noticeably lower than the content of the *Aticl* mutant, which was similar to that of the WT (Fig. 8c, e). Interestingly in rosette leaf, the glucose and fructose contents of the *Aticl* mutant under salt stress were significantly higher than those of the WT, whereas the glucose and fructose contents of the *OsICL*-expressing Arabidopsis lines were more varied and similar to those of the WT (Fig. 8d, f).

## Discussion

The transgenic rice overexpressing *OsCam1–1*, which exhibited higher salt stress tolerance than the WT [18], was shown to have highly increased *OsICL* transcript levels under salt stress, in contrast with *Pinus pinea* seeds, in which ICL activity was decreased under salt stress [22]. This suggested that, in rice, ICL may play important roles in salt tolerance mechanisms. Senescence is known to affect the expression levels of *ICL* genes [23, 24], and we found that *OsICL* was highly expressed in senesced leaf compared with young leaf under the non-stress condition, which is likely under a pre-programmed developmental process. In previous reports, *ICL* was also found to be involved in senescence. For example, Gut and Matile [25] found that *ICL* gene expression was induced in senesced barley leaves, and McLaughlin and Smith [26] found that *ICL* gene expression was induced by acetate under dark condition. In barley leaf and cucumber cotyledon, the abundance of ICL protein increased in the absence of sucrose during darkness-induced senescence in which chlorophyll and protein contents decreased, whereas its abundance was decreased by sucrose supplementation, indicating that ICL and the glyoxylate cycle function in carbohydrate-starved tissues during senescence [27]. The authors suggested that ICL may be involved in the conversion of lipids to organic acids, which are then used in the mobilization of amino acids from leaf proteins. The increase in *OsICL* expression under salt stress that we observed may trigger similar metabolic adjustments during the period of reduced carbon availability.

We found that *OsICL* transcript levels increased in senesced leaf of WT under salt stress, but not in young leaf. Some studies have reported that salt stress can induce plant senescence. For example, Munns [28] reported that toxicity of Na^+^ caused by salt stress resulted in premature senescence, and Lutts et al. [29] found that NaCl affected senescence-related parameters in rice leaf, including chlorophyll fluorescence, membrane permeability, and protein and chlorophyll concentrations. A previous report suggested that CaM1 positively controlled Arabidopsis leaf senescence as leaf yellowing, reactive oxygen species (ROS) accumulation, and expression of *SAG12*, a senescence-associated gene, were enhanced in Arabidopsis overexpressing *CaM1* [30]. Although we found that the *OsICL* upregulation by salt stress in the senesced leaf of the *OsCam1–1*-overexpressing lines was not clearly different from that of the WT, possibly because of their already high expression levels, the *OsICL* expression level was highly up-regulated in the young leaf of the *OsCam1–1* overexpressing lines, but not in the WT. These results suggest that the highly up-regulated *OsICL* expression in young leaf caused by the *OsCam1–1* overexpression may contribute to the salt tolerance phenotype.

The transgenic rice overexpressing *OsCam1–1* reported previously exhibited higher ABA content than the WT under salt stress [18]. The presence of two putative ABREs in the 2-kb upstream sequence of *OsICL* suggests that *OsICL* expression under salt stress was induced by ABA. ABA is a stress hormone in plants that is involved in changes of long-distance transport, stomatal behavior, and gas exchange [31]. ABA controls the transcription of target genes via regulating interaction of transcription factors, including DREB2A/2B, AREB1, and RD22BP1 and MYC/MYB, with their corresponding cis-acting elements, DRE/CRT, ABRE, and MYCRS/MYBRS, respectively [32]. ABA also promotes senescence [33]. Furthermore, in our previous transcriptome study we found that *OsWRKY71* (*LOC_Os02g08440*) expression was induced and enhanced by *OsCam1–1* overexpression [10], which could in turn up-regulate the expression of *OsICL*. This finding supports the presence of the nine putative OsWRKY71 binding regions that were detected in the 2-kb upstream sequence of *OsICL*. Another report in rice aleurone cells showed that *OsWRKY71* was induced by ABA [34]. Together, these results indicate that transcription of *OsICL* may be directly driven by the OsWRKY71 transcription factor, which is influenced by salt-stress-induced senescence, ABA, and *OsCam1–1* expression.

The growth parameters, namely, germination rate, and fresh and dry weights at the seedling stage of the three transgenic *OsICL*-expressing Arabidopsis lines compared with the *Aticl* mutant and the WT showed the impact of OsICL activity on plant growth under salt stress. The highest degree of growth inhibition under salt stress observed in the *Aticl* mutant suggests that ICL affects salt stress tolerance in the germination growth stage. Arabidopsis is an oil seed plant [35], therefore during germination, stored lipid in the seed needs to be converted to an available carbon source for generating energy. Previous studies showed that the *icl* mutant Arabidopsis seedling had defective growth in a non-sucrose supplemented medium, especially under the dark condition, and interestingly, when [^14^C] acetate was supplied to the *icl* mutant line, the amount of ^14^C-labeled sugar in the *icl* mutant line was significantly lower than the WT [11, 12]. This suggests that *ICL* may play a role during germination by using the acetyl unit from acetyl-CoA, the product of stored lipid degradation by β-oxidation, to synthesize sugars via the glyoxylate cycle and gluconeogenesis. A study of two sugar beet hybrids showed that the high vigor hybrid had higher *ICL* transcript levels and higher ICL activity than the low vigor hybrid, especially under salt and H_2_O_2_ stresses, so it was suggested that ICL was a marker for seedling vigor [36]. Together, these results suggest that ICL might play multifunctional roles including salt tolerance, in the plants by modulating energy metabolism.

The results for the vegetative stage further indicate that salt stress affected the *OsICL*-expressing lines to a lesser degree, as indicated by the higher *F*_v_/*F*_m_ values and lower temperature in the leaf, compared with the *Aticl* mutant. Under salt stress, the rosette leaf temperature of the *Aticl* mutant was highest and *F*_v_/*F*_m_ was lowest among the plants examined, which indicated that photosystem II of the *Aticl* mutant was more disrupted. It has been reported that rice leaf temperature measured by infrared thermal imaging was higher when the plant was exposed to high salt concentrations, and the temperature was negatively correlated to stomatal conductance and relative water content [37]. This suggests that the *Aticl* mutant was most affected by salt stress and *OsICL* expression reverted the salt sensitivity phenotype of the *Aticl* mutant. This idea is supported by the phenotype of the *OsICL*-overexpressing line on the WT background, which had the least affected *F*_v_/*F*_m_ value under salt stress.

**Fig. 9:**
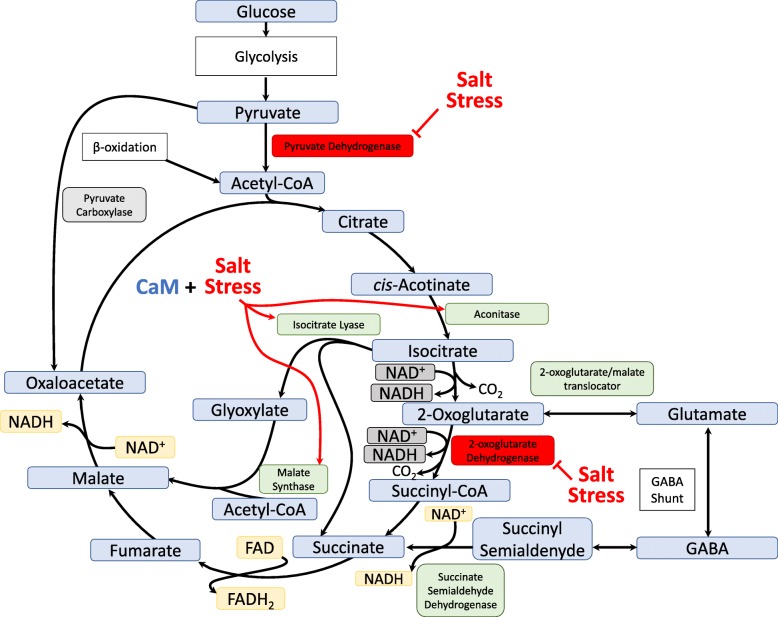
A proposed salt-responsive regulatory mechanism of ICL that can shift energy metabolism from the TCA cycle to the glyoxylate cycle during salt stress. Salt stress in conjunction with CaM induces expression of aconitase, isocitrate lyase and malate synthase genes [10]. In the glyoxylate cycle, ICL generates one NADH from catalyzing malate to oxaloacetate and one FADH_2_ by catalyzing succinate to fumarate bypassing the two decarboxylation steps of the TCA cycle. Two steps of the glyoxylate cycle use the acetyl unit from acetyl-CoA, namely, conversion of glyoxylate to malate and the subsequent conversion of oxaloacetate to citrate. Under salt stress, pyruvate dehydrogenase and 2-oxoglutarate dehydrogenase complex are inhibited [39]. The respiratory metabolism was previously proposed to be shifted to the GABA shunt pathway to provide alternative carbon source [39]. Because the pyruvate dehydrogenase complex is sensitive to salt stress, pyruvate might instead be fluxed to pyruvate carboxylase to generate oxaloacetate. Additionally, as 2-oxoglutarate dehydrogenase complex is inhibited under salt stress, the disrupted TCA cycle could lead to metabolite flux into the glyoxylate cycle to bypass the salt-inhibited enzymes

Figure 9 describes a proposed salt-responsive regulatory mechanism of ICL that can shift energy metabolism from the TCA cycle to the glyoxylate cycle during salt stress. In the glyoxylate cycle, ICL generates succinate and glyoxylate, which is the precursor for malate. The catalysis of succinate to fumarate generates one FADH_2_ and the catalysis of malate to oxaloacetate generates one NADH. The glyoxylate cycle bypasses the two decarboxylation steps of the TCA cycle. Two steps of the glyoxylate cycle use the acetyl unit from acetyl-CoA, namely, the conversion of glyoxylate to malate and the subsequent conversion of oxaloacetate to citrate. Acetyl-CoA can be generated by β-oxidation or catabolism of some amino acids, namely, leucine, isoleucine, valine, alanine, serine, and cysteine [38]. A previous report in wheat revealed that the TCA cycle, which is a major respiratory process, was inhibited by salt stress and that the pyruvate dehydrogenase and 2-oxoglutarate dehydrogenase complexes were salt sensitive; therefore, the respiratory metabolism was shifted to the GABA shunt pathway to provide an alternative carbon source [39]. In our previous study in rice, we found that the expression levels of genes encoding aconitase and malate synthase were up-regulated by the effect of salt stress and *OsCam1–1* overexpression [10]. Because the pyruvate dehydrogenase complex was sensitive to salt stress, we speculated that pyruvate might instead be fluxed to pyruvate carboxylase to generate oxaloacetate. Additionally, 2-oxoglutarate dehydrogenase complex might be inhibited under salt stress. Therefore, we propose that the disrupted TCA cycle could lead to metabolite flux into the glyoxylate cycle to bypass the salt-inhibited enzymes.

The glyoxylate cycle generates NADH and FADH_2_ to be fed into the electron transport chain to generate energy. Therefore, lacking the important activity of ICL would disrupt the metabolic flux of the glyoxylate cycle as mentioned above, and with the inhibition of pyruvate dehydrogenase and 2-oxoglutarate dehydrogenase under salt stress, this would presumably result in glucose and fructose being inefficiently fed into the metabolic flux of the central energy metabolism process. We found that the glucose and fructose contents of the *Aticl* mutant, which was the most salt-sensitive line, were highest among the five Arabidopsis plants examined, and the *OsICL* expression reverted it. Together, these results provide novel evidence to support the role of ICL, the key enzyme in the glyoxylate cycle, in plant salt tolerance.

## Conclusions

This study suggests that, in rice, *OsICL* plays roles under salt stress through acclimation of energy metabolism and that *OsCam1–1* may be involved in regulating its transcription. The role of *OsICL*, a salt-responsive gene was characterized in transgenic Arabidopsis revealing that *ICL* expression reverted the salt sensitivity phenotypes as well as the glucose and fructose contents of the *Aticl* mutant. Together, these results suggest that ICL may facilitate the shift of energy metabolism to the glyoxylate cycle to modulate carbon balance and provide necessary energy during salinity stress, thereby contributing to salt tolerance in plants.

## Methods

### Plant materials and growing conditions

Three lines of transgenic Khao Dawk Mali 105 (salt-sensitive) rice overexpressing *OsCam1–1* [18] were grown hydroponically in Yoshida solution [40] for 3 weeks with completely randomized design. Then, the rice was treated with 150 mM NaCl for 4 h. The seedlings were collected and snap frozen in liquid nitrogen. Seeds of the *Aticl* Arabidopsis mutant (GK-008E03) were obtained from Nottingham Arabidopsis Stock Centre (UK). The seed was decontaminated by quickly rinsing with 75% ethanol and soaking in 2% NaOCl for 10 min before rinsing with sterilized water containing Tween 80 for 5–8 times, and then transferred to a Murashige & Skoog (MS) medium (PhytoTechnology Laboratories®, USA) plate with 1% w/v sucrose. The seed was sown on the plate and stored at 4 °C for 2 days. Then the plate was moved to a growth chamber or a growth room at 25 °C under 16-h/8-h light/dark period for 1 week. The plant was transferred to a pot containing planting material, peat moss:perlite:vermiculite in a 3:1:1 ratio, and grown under the same condition, and watered every 2–3 days until the plant matured. The seeds were harvested and stored at 4 °C.

### qRT-PCR analysis

Plant leaf samples were ground with liquid nitrogen using a chilled mortar and pestle until they became fine powder. Total RNA was extracted using TRI-Reagent® (Molecular Research Center, USA) following the manufacturer’s protocol, then the RNA was dissolved in diethylpyrocarbonate-treated water. The quantity and quality of the extracted RNA were checked by a spectrophotometric method and agarose gel electrophoresis. The RNA was treated with RNAse-free DNase I (Thermo Fisher, USA) and converted to cDNA using an iScript™ cDNA Synthesis Kit (Bio-Rad, USA). The qRT-PCRs were performed using SsoFast™ EvaGreen® Supermixes (Bio-Rad, USA). The sequences and PCR conditions of the primers used in this study are shown in Additional file 3. Gene expression levels were calculated employing the 2^-(ΔΔCT)^ and 2^-(ΔCT)^ methods with *EF1-α* as the internal control. For the rice gene expression analysis, four biological replicates were used, and for the Arabidopsis gene expression analysis, five biological replicates were used.

### Construction of transgenic Arabidopsis expressing OsICL

Two recombinant plasmids were constructed to express *OsICL* in Arabidopsis, namely, p*AtICL*-*OsICL*-pK2GW7, which was designed to drive *OsICL* expression using the native *AtICL* promoter, and p35SCaMV-*OsICL*-pK2GW7, which was designed to drive *OsICL* overexpression using the 35 cauliflower mosaic virus (35SCaMV) promoter. To construct those recombinant plasmids, three DNA fragments, namely, β-glucuronidase and nopaline synthase terminator (GUS-NOS) from pCAMBIA1301, the 2138-bp upstream sequence of the Arabidopsis isocitrate lyase gene (p*AtICL*) from the Arabidopsis genomic DNA, and the rice isocitrate lyase (*OsICL*) coding sequence from the japonica rice cDNA library (AK063353, KOME clone number 001–114-C03), were amplified by PCR. First, the DNA fragment of the *OsICL* coding sequence was engineered with an *Nde*I restriction site on the 5′ end using primers *Nde*I_*OsICL*_F and *OsICL*_R (Additional file 4), before cloning into pTZ57R/T, a TA cloning plasmid (Thermo Fisher, USA), using blue-white colony screening on an ampicillin plate, resulting in *OsICL-*pTZ57R/T. The p*AtICL* sequence was engineered with *Xba*I and *Nde*I restriction sites using primers *Xba*I_p*AtICL*_F and p*AtICL*_*Nde*I_R (Additional file 4), and cloned into *OsICL-*pTZ57R/T at the *Xba*I and *Nde*I restriction sites. The resulting p*AtICL*-*OsICL*-pTZ57R/T was double-digested by *EcoR*I and *Xba*I. The GUS-NOS fragment was engineered with *EcoR*I and *Xba*I restriction sites using primers; *EcoR*I_DTOPO_GUS-NOS_F and GUS-NOS_*Xba*I_R (Additional file 4), and then cloned into the p*AtICL*-*OsICL*-pTZ57R/T, resulting in the recombinant plasmid, GUS-NOS-p*AtICL*-*OsICL*-pTZ57R/T. Then, a cassette of GUS-NOS-p*AtICL*-*OsICL* was amplified from this recombinant plasmid using DTOPO_GUS-NOS_F and *OsICL*_R primers (Additional file 4). The GUS-NOS-p*AtICL*-*OsICL* fragment was inserted into a pENTR/D-TOPO gateway directional cloning plasmid (Invitrogen™, USA). The cassette of GUS-NOS-p*AtICL*-*OsICL* in pENTR/D-TOPO was cloned to a pK2GW7 vector by Gateway cloning using LR clonase II (Invitrogen, USA). This recombinant plasmid was designed to express *OsICL* under the native *AtICL* promoter. Two other plasmids designed to overexpress *OsICL* and *OsCam1–1* were constructed using the *OsICL* fragment, which was added with CACC in front of the start codon using primers, DTOPO_*OsICL*_F and *OsICL*_R (Additional file 4). The fragment was directionally cloned into a pENTR-DTOPO vector, then further cloned into a pK2GW7 vector by Gateway cloning. Therefore, *OsICL* should be driven by the 35SCaMV promoter on the pK2GW7 vector. The DNA sequences of the recombinant plasmids, GUS-NOS-p*AtICL*-*OsICL-*pK2GW7 (Additional file 5), and OX*OsICL-*pK2GW7 (Additional file 6) were determined. *Agrobacterium tumefaciens* GV3101 was transformed with individual plasmids and used for floral dipping [41] of the *Aticl* Arabidopsis mutant to generate Arabidopsis lines expressing *OsICL* under a 2138-bp upstream sequence of *AtICL* (3FL9), and overexpressing *OsICL* (OX*OsICL*/*icl*). *A. tumefaciens* was also transformed with OX*OsICL-*pK2GW7 and used for floral dipping of the WT Arabidopsis to generate the *OsICL* (OX*OsICL*/WT) overexpressing line.

The floral-dipped Arabidopsis seeds (T1) were selected on 1% w/v sucrose MS medium with 50–100 μg/ml kanamycin. Survival rate of the T2 seeds were determined by germinating 30–100 seeds from each T1 plant in kanamycin. The expected survival rate of the hemizygous T2, which has no allelic counterpart, was 75%. The χ^2^ test was applied and statistical significance was set at *p* < 0.05. The T2 seeds that exhibited the accepted survival rate of 3:1 were grown further to obtain the offspring seeds (T3). The T3 seeds were grown on the MS medium with kanamycin to find homozygous lines among the mixture of hemizygous and homozygous plants. Therefore, survival rate of 100% was expected for the homozygous plants.

### Genotyping of the transgenic Arabidopsis plants by PCR

Genomic DNA was extracted by grinding plant leaf (around 100 mg) using a Mixer Mill MM 400 (Retsch, Germany) in a 1.5-ml microtube. Then, 300 μl of plant genomic extraction buffer containing 200 mM Tris buffer (pH 7.5), 25 mM EDTA, 250 mM NaCl, and 0.05% w/v SDS was mixed with the samples. Equal volume of 99% isopropanol was added to the sample mixture and mixed. The mixture was centrifuged at 12,000×g for 15 min, then the supernatant was discarded. The pellet was dried at room temperature for about 15 min or until the isopropanol completely evaporated. Then, 50 μl of sterilized distill water was added to dissolve the pellet.

The mutant was confirmed by PCR genotyping using three primers, LP, RP, and LB (Additional file 7). LP and RP were located on the gene and LB was located on the inserted T-DNA on the 4th exon of At*ICL* (Additional file 8). The PCR product using the LP and RP primers should be about 911 bp in Arabidopsis without the T-DNA insertion (WT), whereas the PCR product using the LB and RP primers should be about 500 bp in Arabidopsis containing T-DNA-inserted *AtICL* (*Aticl* mutant). The inserted T-DNA in the mutant is sufficiently long to obstruct PCR amplification using the LP and RP primers. In the heterozygous plants, two PCR products of 911 bp and about 500 bp should be expected; therefore, one more generation was needed to select homozygous plants to obtain the mutant for further experiments.

PCR genotyping employing specific primer OsICL_Seq_M3_F (Additional file 7), which is located on the coding region of *OsICL*, and 35S_Terminator_Seq_R (Additional file 7) was conducted to confirm *OsICL* insertion into the transgenic Arabidopsis. The PCR product size of the transgenic Arabidopsis expressing *OsICL* was expected to be about 460 bp.

### Isocitrate lyase activity assay

Approximately 50 mg of total plant leaf tissues from the five Arabidopsis lines, 3FL9, OX*OsICL*/*icl*, OX*OsICL*/WT, *Aticl* mutant, and WT, grown in 1% w/v sucrose MS medium for 10 days from three biological replicates were collected, frozen in liquid nitrogen and ground to a fine powder using a Mixer Mill. Then, 500 μl of extraction buffer (100 mM potassium phosphate buffer at pH 7.6, 10 mM MgCl_2_, 1 mM EDTA, and 1 mM DTT) [22] was added to the ground samples and mixed by vortex. The mixture was centrifuged at 12,000×g for 20 min at 4 °C. The supernatant was transferred to a new microtube for use as crude protein extract in the enzymatic activity assay and the pellet was discarded.

Next, 50 μl of extracted protein was added into 440 μl of reaction buffer containing 50 mM potassium phosphate buffer at pH 6.9, 50 mM MgCl_2_, 10 mM EDTA, and 40 mM phenylhydrazine, which was modified from Cooper and Beevers [15], then mixed at 30 °C using a pipette in a 1-ml quart cuvette with 1-cm path length. The absorbance of the reaction mixture at A_324_ was measured by spectrophotometer until it was stable, then 10 μl of 500 mM D-L isocitric acid was added to the mixture and homogeneously mixed. Then, the absorbance of the reaction mixture at A_324_ was measured for 10 min. The difference between the 0 min and 10 min A_324_ values was used to calculate the amount of glyoxylate production per 10 min using the glyoxylate standard curve. The protein content of an aliquot of the crude protein extract was determined using Bradford reagent in a 96-well plate. Absorbance at A_595_ was measured using a microplate reader Synergy H1 (Biotek®, USA), and bovine serum albumin was used to construct a standard curve.

### Growth measurements

Seeds of the five Arabidopsis lines; 3FL9, OX*OsICL*/*icl*, OX*OsICL*/WT, *Aticl* mutant and WT were decontaminated as described above. Then, the seeds were orderly sown onto MS medium with 0, 100, 120, 150, or 200 mM NaCl. The Arabidopsis plates were stored at 4 °C for 2 days. The number of germinated seeds was observed daily for 7 days after the plates were moved to the growing condition. We performed five biological replicates, and each biological replicate contained 50 seeds of each line. The germination rate was reported as percentage with standard deviation.

Following these plant growing steps, the growing period was extended to 10 days. Then, 10 seedlings of each biological replicate of the *Aticl* mutant and WT Arabidopsis, 3FL9, OX*OsICL*/*icl*, and OX*OsICL*/WT were weighed to determine the fresh weight. The samples were then baked at 60–70 °C in a hot air oven for 5–7 days. The baked samples were weighed to determine the dry weight.

### Leaf F_v_/F_m_ and temperature measurement

The 4-week-old vegetative stage of the five Arabidopsis lines, 3FL9, OX*OsICL*/*icl*, OX*OsICL*/WT, *Aticl* mutant, and WT, growing in pots containing 3:1:1 of peat moss:perlite:vermiculite were treated with 300 mM NaCl for 3 days. Then dark adaptation was allowed for 30 mins before the *F*_v_*/F*_m_ was measured under the dark condition using a pocket PEA fluorometer (Hansatech, UK). Rosette leaf temperature was measured using a FLIR C2 thermal camera (FLIR, USA). For *F*_v_*/F*_m_, four biological replicates were used, and for leaf temperature, six biological replicates were used.

### Sugar content measurements

The 4-week-old vegetative stage of the five Arabidopsis lines, 3FL9, OX*OsICL*/*icl*, OX*OsICL*/WT, *Aticl* mutant, and WT, growing in pots containing 3:1:1 of peat moss:perlite:vermiculite from six biological replicates were treated with 300 mM NaCl for 3 days. The rosette leaves used were collected, frozen in the liquid nitrogen, and lyophilized. The dry weight was measured and the samples were ground to fine powder. The samples were extracted in deionized water and filtered. Glucose, sucrose and fructose contents were determined using high performance liquid chromatography (HPLC) (Shimadzu, Japan) with Hi-Plex Ca (Duo) column (Agilent, USA). The HPLC flow rate was 0.25 ml/min, column temperature was 85 °C, 100% ultra-pure water was used as the mobile phase, and a refractive index detector (RID) was used to detect the sugars.

### Statistical analysis

Data from q RT-PCRs, enzyme activity assays, and growth parameters were compared using analysis of variance (ANOVA), and the means were compared with Duncan’s multiple range test, with significance set as *p* < 0.05. The germination rate data were compared using ANOVA, and the means were compared with Duncan’s multiple range test, with significance set as *p* < 0.01.

## Supplementary information

**Additional file 1. **A) PCR amplification verifying the cloning of *GUS-NOS*, *AtICL* upstream sequence, and *OsICL* coding sequence into the recombinant plasmid *GUS-NOS*-upstream-*AtICL-OsICL*-pK2GW7: lane M, DNA marker; lane 1, PCR product of the cloned *GUS-NOS* with size of around 2300 bp; lane 2, PCR product of the cloned *AtICL* upstream sequence with size of around 2100 bp; lane 3, PCR product of the cloned *OsICL* coding sequence with size of around 1700 bp. B) PCR amplification verifying the insertion of *OsICL* coding sequence in the recombinant plasmid *OsICL*-pK2GW7: lane M, DNA marker; lane 1, PCR product of the cloned *OsICL* coding sequence with size of around 1700 bp. C) PCR genotyping of the transgenic Arabidopsis background: lane M, DNA marker; lane 1 *aticl* mutant; lane 2 wild type; lane 3 3FL9; lane 4 OX*OsICL*/*icl*; lane 5 OX*OsICL*/WT. D) PCR amplification verifying *OsICL* gene insertion: lane M, DNA marker; lane 1 *aticl* mutant; lane 2 wild type; lane 3 3FL9; lane 4 OX*OsICL*/*icl*; lane 5 OX*OsICL*/WT. The PCR products were analyzed by agarose gel electrophoresis using TAE buffer with 1% agarose gel under 100 mV for 30 min.

**Additional file 2.** Thermograms of the five Arabidopsis lines from FLIR C2 thermal camera.

**Additional file 3.** Nucleotide sequences and PCR conditions of the primers used for qRT-PCR.

**Additional file 4. **Nucleotide sequences of the primers used for construction of the recombinant plasmids for expressing *OsICL*. The bold characters represent restriction sites, the italic characters represent the added nucleotides benefitting in binding of restriction enzyme, and the underlined characters represent the sequence benefitting in directional TOPO cloning.

**Additional file 5. **Schematic diagrams showing construction of the recombinant plasmid for expressing *OsICL* under the control of *AtICL* promoter. A) the three fragments were inserted into pTZ57R/T employing restriction site cloning strategy, and the target fragments were amplified from the recombinant plasmid GUS-NOS-p*AtICL*-*OsICL*-pTZ57R/T by PCR. B) The GUS-NOS-p*AtICL*-*OsICL* cassette was directionally inserted into pENTR/DTOPO employing “CACC” site, then it was subcloned into pK2GW7, the destination vector, by Gateway cloning strategy.

**Additional file 6. **Schematic diagrams showing construction of the recombinant plasmid for overexpressing *OsICL* employing directional TOPO and Gateway cloning strategies.

**Additional file 7. **Nucleotide sequences of the primers used for PCR genotyping the wild type, the *aticl* mutant, and the transgenic Arabidopsis lines.

**Additional file 8. **The inserted T-DNA location on the *icl* Arabidopsis mutant (GK-008E03) and the positions of the primers for *icl* Arabidopsis mutant genotyping: LP and RP represent primers locating on the *AtICL* gene and LB represents primer locating on the inserted T-DNA.

## Data Availability

All relevant data are included in this article and its Additional files.

## Abbreviations

ABA: Abscisic acid
*AtICL*: *Arabidopsis thaliana* isocitrate lyase gene
CaM: Calmodulin
ICL: Isocitrate lyase
*OsCam1–1*: *Oryza sativa* L. calmodulin 1–1 gene
*OsICL*: *Oryza sativa* L. isocitrate lyase gene
TCA: Tricarboxylic acid cycle
WT: Wild type
