# Supplementary figures and images for: Isocitrate lyase plays important roles in plant salt tolerance
Source: BMC Plant Biol. 2019 Nov 6;19:472. doi: 10.1186/s12870-019-2086-2 (PMC6833277; doi:10.1186/s12870-019-2086-2)

# Additional File 1

**A**

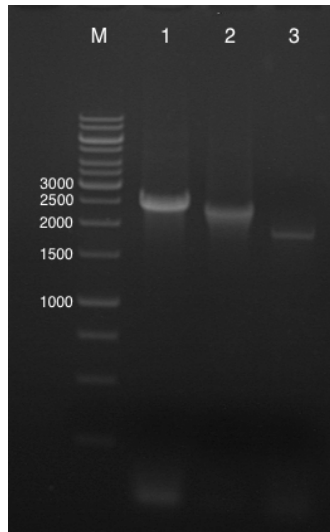

**B**

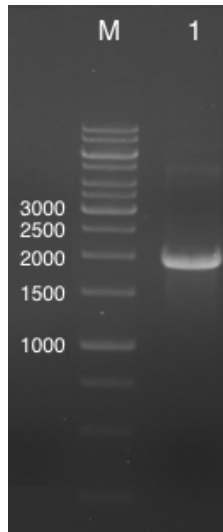

**C**

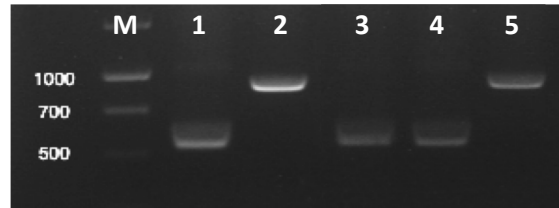

**D**

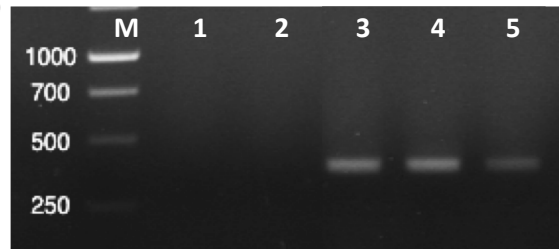

Supplement: Supplementary file 1 — Additional file 1. A) PCR amplification verifying the cloning of GUS-NOS, AtICL upstream sequence, and OsICL coding sequence into the recombinant plasmid GUS-NOS-upstream-AtICL-OsICL-pK2GW7: lane M, DNA marker; lane 1, PCR product of the cloned GUS-NOS with size of around 2300 bp; lane 2, PCR product of the cloned AtICL upstream sequence with size of around 2100 bp; lane 3, PCR product of the cloned OsICL coding sequence with size of around 1700 bp. B) PCR amplification verifying the insertion of OsICL coding sequence in the recombinant plasmid OsICL-pK2GW7: lane M, DNA marker; lane 1, PCR product of the cloned OsICL coding sequence with size of around 1700 bp. C) PCR genotyping of the transgenic Arabidopsis background: lane M, DNA marker; lane 1 aticl mutant; lane 2 wild type; lane 3 3FL9; lane 4 OXOsICL/icl; lane 5 OXOsICL/WT. D) PCR amplification verifying OsICL gene insertion: lane M, DNA marker; lane 1 aticl mutant; lane 2 wild type; lane 3 3FL9; lane 4 OXOsICL/icl; lane 5 OXOsICL/WT. The PCR products were analyzed by agarose gel electrophoresis using TAE buffer with 1% agarose gel under 100 mV for 30 min. [file 12870_2019_2086_MOESM1_ESM.pdf]

Additional File 2

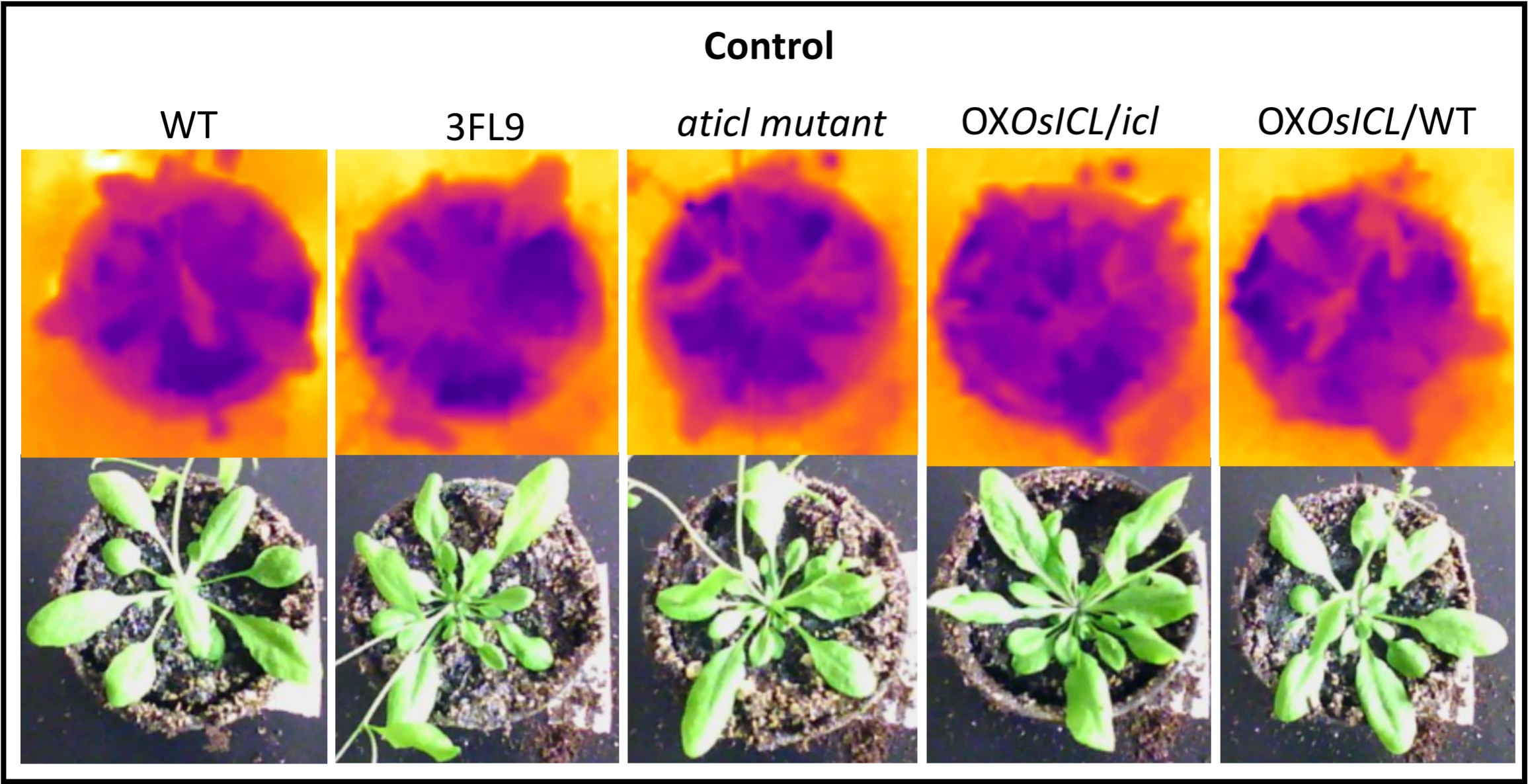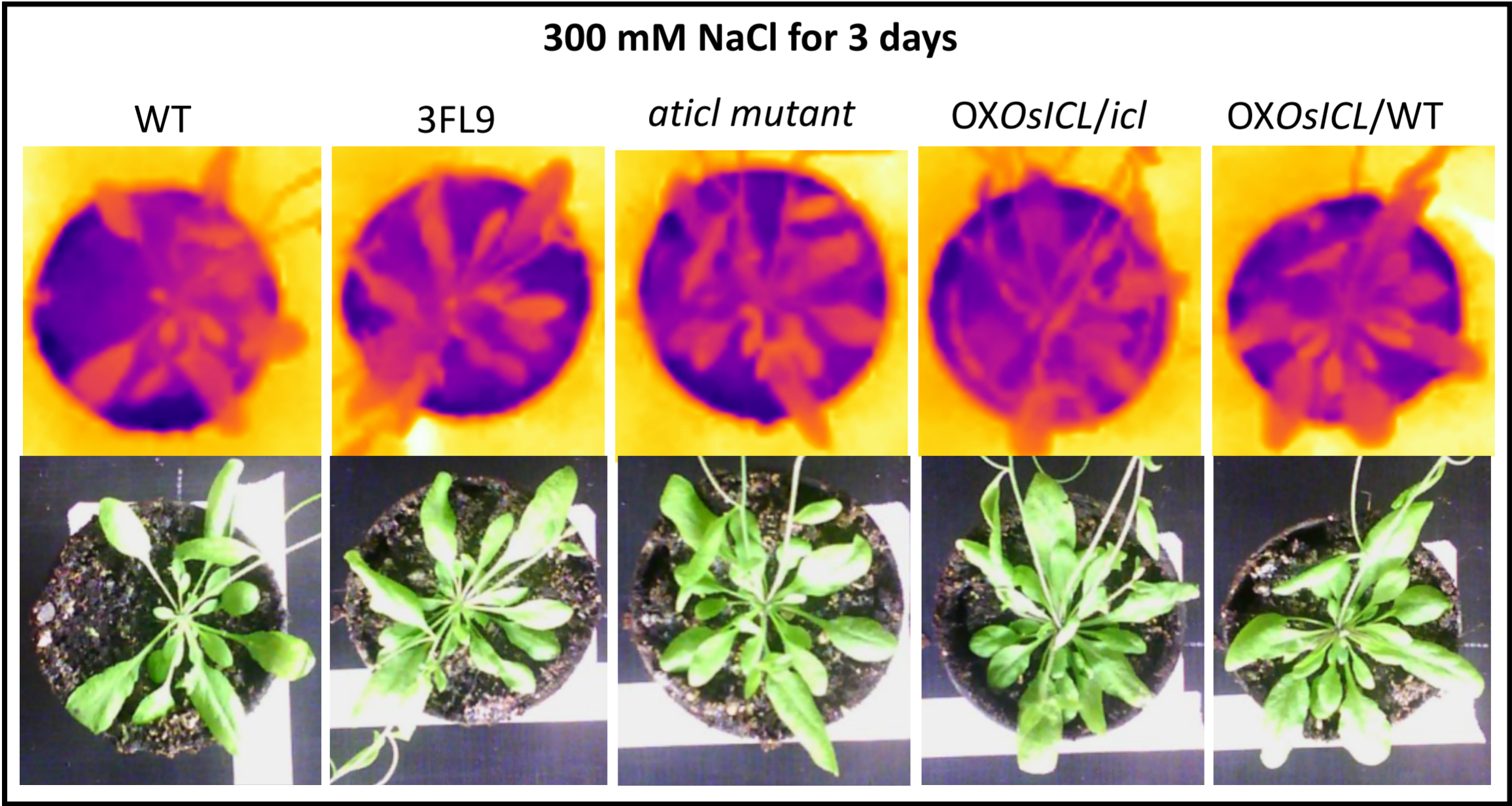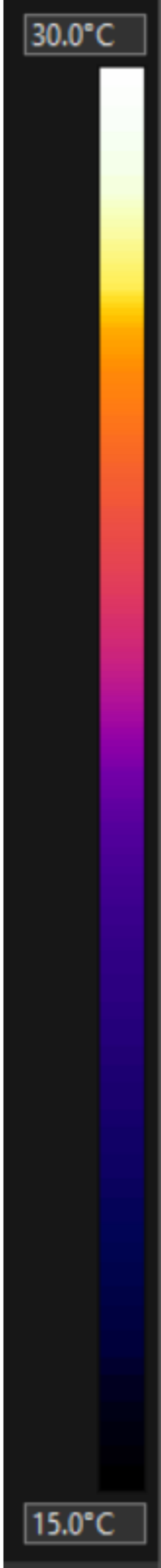

Supplement: Supplementary file 2 — Additional file 2. Thermograms of the five Arabidopsis lines from FLIR C2 thermal camera. [file 12870_2019_2086_MOESM2_ESM.pdf]

## Additional File 5

A

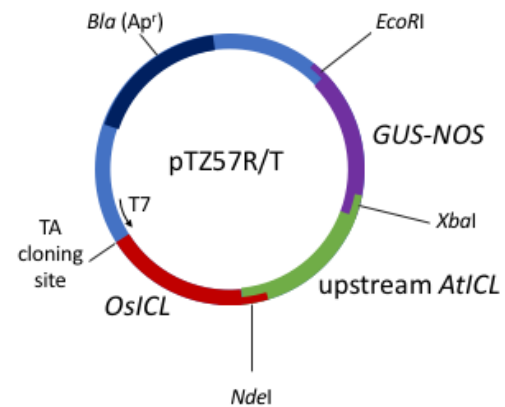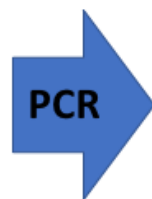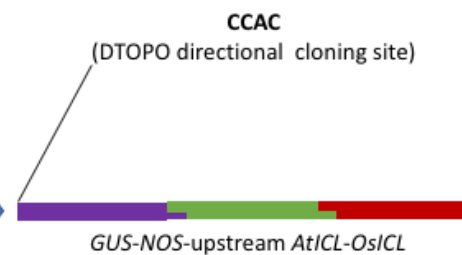

B

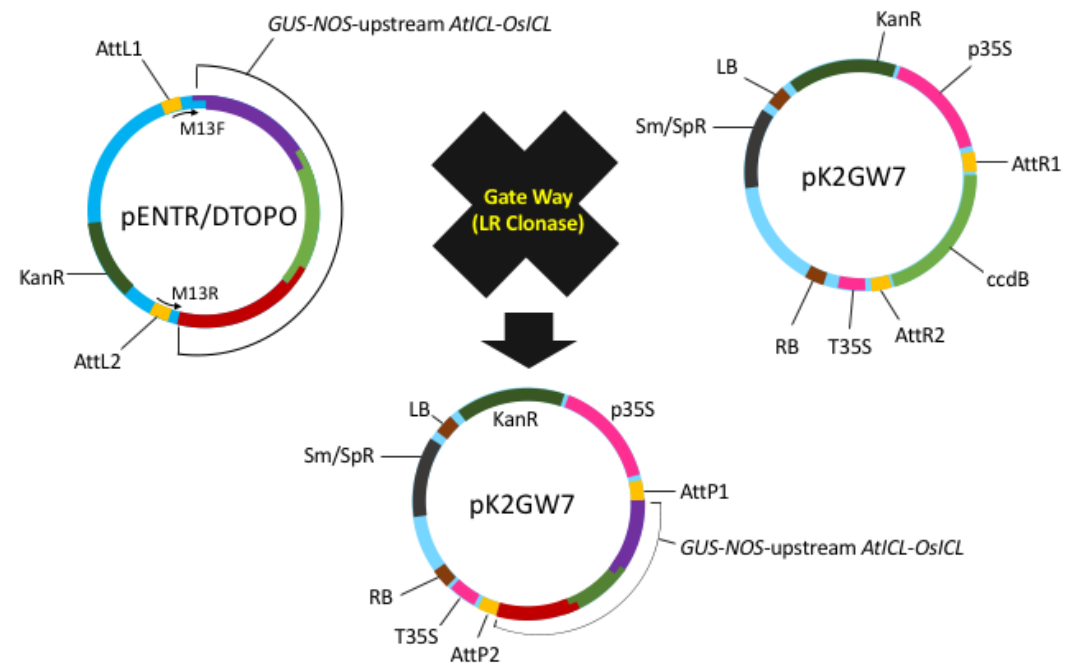

Supplement: Supplementary file 5 — Additional file 5. Schematic diagrams showing construction of the recombinant plasmid for expressing OsICL under the control of AtICL promoter. A) the three fragments were inserted into pTZ57R/T employing restriction site cloning strategy, and the target fragments were amplified from the recombinant plasmid GUS-NOS-pAtICL-OsICL-pTZ57R/T by PCR. B) The GUS-NOS-pAtICL-OsICL cassette was directionally inserted into pENTR/DTOPO employing “CACC” site, then it was subcloned into pK2GW7, the destination vector, by Gateway cloning strategy. [file 12870_2019_2086_MOESM5_ESM.pdf]

## Additional file 6

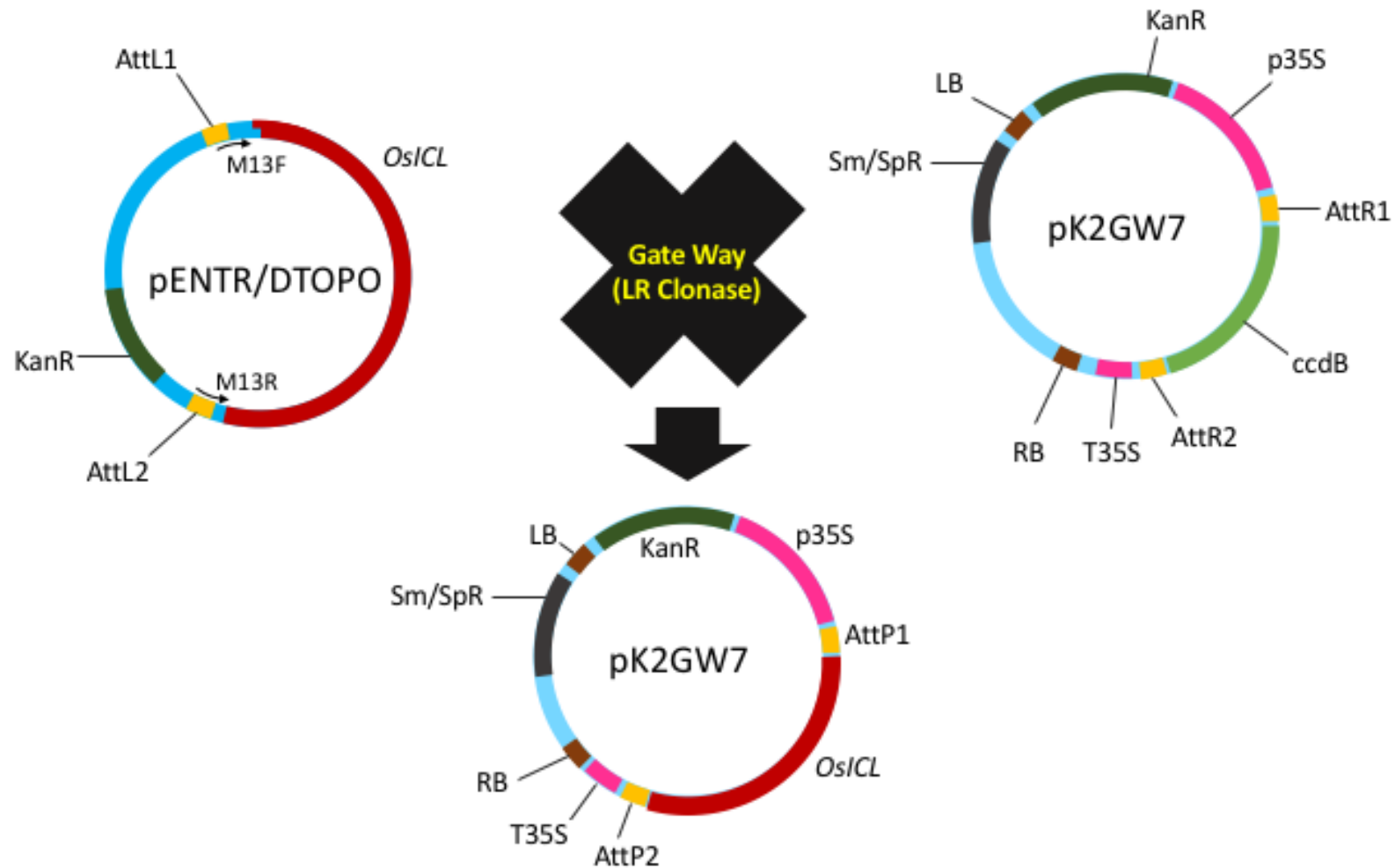

Supplement: Supplementary file 6 — Additional file 6. Schematic diagrams showing construction of the recombinant plasmid for overexpressing OsICL employing directional TOPO and Gateway cloning strategies. [file 12870_2019_2086_MOESM6_ESM.pdf]

## Additional File 8

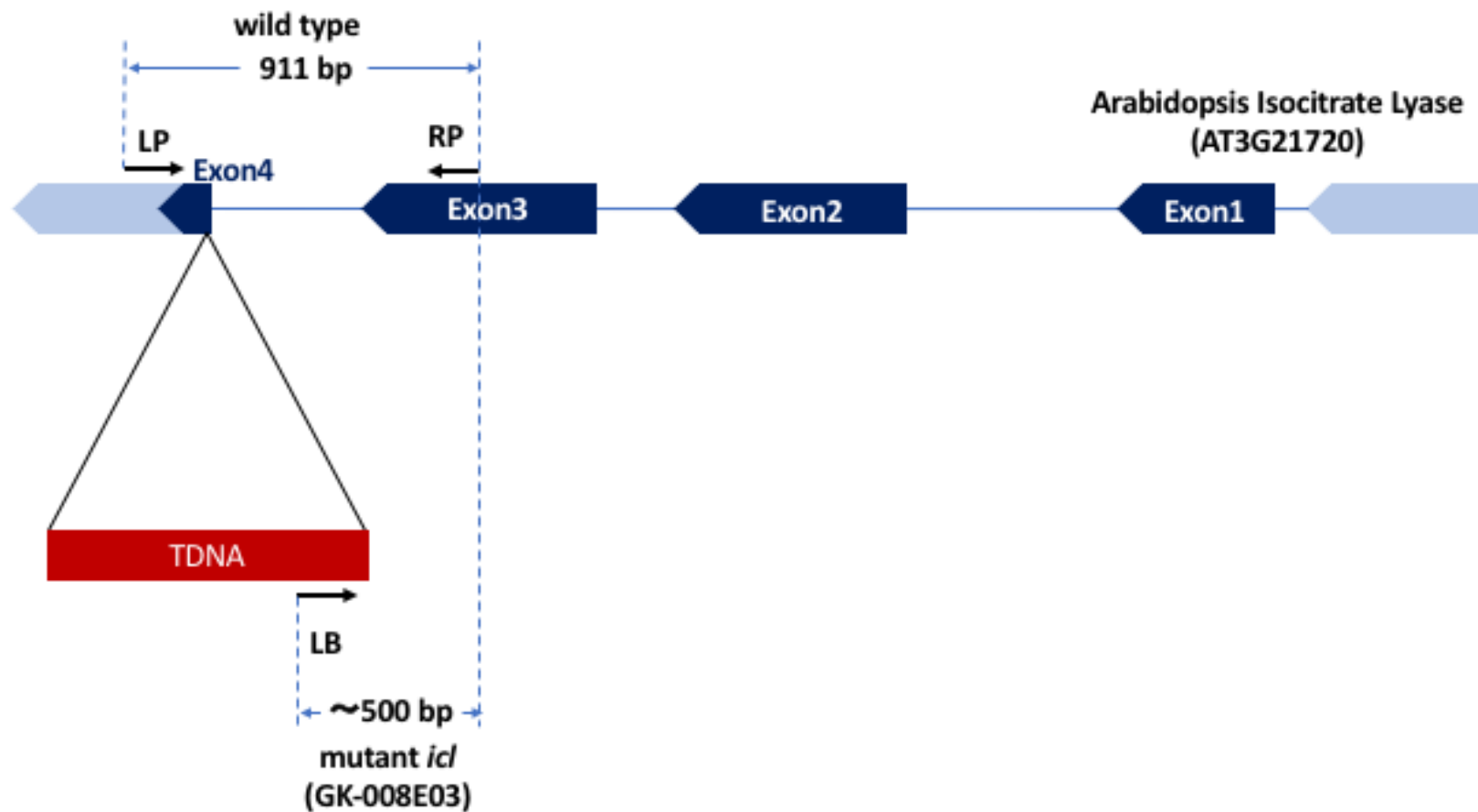

Supplement: Supplementary file 8 — Additional file 8. The inserted T-DNA location on the icl Arabidopsis mutant (GK-008E03) and the positions of the primers for icl Arabidopsis mutant genotyping: LP and RP represent primers locating on the AtICL gene and LB represents primer locating on the inserted T-DNA. [file 12870_2019_2086_MOESM8_ESM.pdf]
