# Supplementary material for: Isocitrate lyase plays important roles in plant salt tolerance
Source: BMC Plant Biol. 2019 Nov 6;19:472. doi: 10.1186/s12870-019-2086-2 (PMC6833277; doi:10.1186/s12870-019-2086-2)
Supplement: Supplementary file 3 — Additional file 3. Nucleotide sequences and PCR conditions of the primers used for qRT-PCR. [file 12870_2019_2086_MOESM3_ESM.pdf]

Additional File 3

Gene	Primer sequence	Locus Number	Efficiency	R^2	Tm	PCR Product Size (bp)
Rice						
OsEF1-α	F-5′ATGGTTGTGGAGACCTTC3′<br>R-5′TCACCTTGGCACCGGTTG3′	LOC_Os03g08010	94.9	0.977	55.0	218
isocitrate lyase	F-5′AGAGCAGCAGCCATGTTCTT3′<br>R-5′CGTGCGTGCTGTAGTTCAGT3′	LOC_Os07g34520	90.3	0.990	59.6	150
Arabidopsis						
AtEF1-α	F-5′TTCGCTGTTAGGGACATGAGGC3′<br>R-5′CACCCTTCTTCACTGCAGCCTT3′	AT1G07930	105.6	0.955	56.6	109
Isocitrate lyase (*AtICL*)	F-5′ TCTACGTGGCCATCTCAAGC3′<br>R-5′ TGGCTTGATGGCTTTTGAGC3′	AT3G21720	100.7	0.955	61.5	83
Isocitrate lyase (*OsICL*) (inserted *OsICL* CDS)	F-5′ AGAGGGAGGAGAGGAGCAAC3′<br>R-5′ CCTGGATTTGGCAAGAACAT3′	LOC_Os07g34520	107.5	0.976	56.6	212
