# Supplementary material for: Isocitrate lyase plays important roles in plant salt tolerance
Source: BMC Plant Biol. 2019 Nov 6;19:472. doi: 10.1186/s12870-019-2086-2 (PMC6833277; doi:10.1186/s12870-019-2086-2)
Supplement: Supplementary file 4 — Additional file 4. Nucleotide sequences of the primers used for construction of the recombinant plasmids for expressing OsICL. The bold characters represent restriction sites, the italic characters represent the added nucleotides benefitting in binding of restriction enzyme, and the underlined characters represent the sequence benefitting in directional TOPO cloning. [file 12870_2019_2086_MOESM4_ESM.pdf]

Additional File 4

Primer Name	Sequence (5' to 3')
*Nde*I_*OsICL*_F	*GTT***CATATG**TCGTCGCCGTTCTCCGTGCCAT
*OsICL*_R	TCACATCCTGGATTTGGCAAGA
*Xba*I_p*AtICL*_F	*A***TCTAGA**CATCATCTTCTATCGGAATCTCA
p*AtICL*_*Nde*I_R	*TGG***CATATG**TTTAACTTTTATAAATTGGAAATG
*Eco*RI_DTOPO_GUS-NOS_F	*TAGAT***GAATTC**ACCATGGTAGATCTGAGGGTAAATTTCTAG
GUS-NOS_*Xba*I_R	*T***TCTAGA**ATAATTTATCCTAGTTTGCGCGCTA
DTOPO_GUS-NOS_F	CACCATGGTAGATCTGAGGGTAAATTTCTAG
DTOPO_*OsICL*_F	CACCATGTCGTCGCCGTTCTCCGTGCCAT
T7 promoter	TAATACGACTCACTATAGGG
M13R	CAGGAAACAGCTATGAC
35S_terminator_R	CTAGCATGGCCGCGGATAT
