# Supplementary material for: Isocitrate lyase plays important roles in plant salt tolerance
Source: BMC Plant Biol. 2019 Nov 6;19:472. doi: 10.1186/s12870-019-2086-2 (PMC6833277; doi:10.1186/s12870-019-2086-2)
Supplement: Supplementary file 7 — Additional file 7. Nucleotide sequences of the primers used for PCR genotyping the wild type, the aticl mutant, and the transgenic Arabidopsis lines. [file 12870_2019_2086_MOESM7_ESM.pdf]

Additional File 7

Primer Name	Sequence (5' to 3')
LP	5'GGTAAGAGTCTATCGGGCTTAGTG3'
RP	5'GTTATCTTAAGACCGTCCAAGGTG3'
LB	5'ATAATAACGCTGCGGACATCTACATTTT3'
OsICL_Seq_M3_F	5’TGTGGCAGTTCATCACGCTC3’
35S_Terminator_Seq_R	5’CTAGCATGGCCGCGGGATAT3’
